# New mosquito repellency bioassay for evaluation of repellents and pyrethroids using an attractive blood-feeding device

**DOI:** 10.1186/s13071-021-04656-y

**Published:** 2021-03-10

**Authors:** Yasue Morimoto, Hitoshi Kawada, Kan-ya Kuramoto, Takuya Mitsuhashi, Toshinobu Saitoh, Noboru Minakawa

**Affiliations:** 1 Graduate School of Biomedical Sciences, Nagasaki University, Nagasaki, Japan; 2 Institute of Tropical Medicine, Nagasaki University, Nagasaki, Japan; 3 Overseas Standards Testing Laboratory, Kaken Test Center, Tokyo and Osaka, Japan

**Keywords:** DEET, Mosquito, Permethrin, Repellency, Test method

## Abstract

**Background:**

With the increasing threat of the worldwide spread of mosquito-borne infectious diseases, consumer interest in anti-mosquito textiles that protect against mosquito bites is also increasing. Accordingly, repellent- or insecticide-treated textiles are gaining popularity. The standardization of commercial textile products is, therefore, indispensable for an authentic and objective evaluation of these products. Here we report a textile testing method using an artificial blood-feeding system that does not involve human volunteers or live animals, which aligns with the policy of protecting human and animal welfare.

**Methods:**

The attractive blood-feeding device (ABFD) was designed using the Hemotek® membrane feeding system. The repellency of DEET, icaridin and permethrin was assayed using unfed female adults of *Aedes albopictus* (Skuse) under two different test conditions, namely choice and no-choice tests. The choice test consisted of two feeding units, one chemically treated and untreated, that were installed on the ABFD; mosquitoes attracted to and resting on the feeding units were counted and the overall blood-feeding rates recorded. The no-choice test consisted of two feeding units treated with the same chemical that were installed on the ABFD; mosquitoes attracted to and resting on the feeding units were counted and the blood-feeding rates were recorded. A control test was conducted using two feeding units, both sides of which were untreated.

**Results:**

In the choice test, high repellency (> 95% inhibition of resting on the treated surface) of 1% DEET and 2% icaridin was observed, whereas 2% permethrin was not an effective repellent. Also, high blood-feeding inhibition (> 95%) was observed for 2% DEET and 2% icaridin. In the no-choice test, high repellency was observed for 1% DEET and 2% icaridin, whereas the repellency of 2% permethrin was low. Also, high blood-feeding inhibition was observed for 2% DEET, 4% icaridin and 2% permethrin.

**Conclusions:**

The accuracy and reproducibility of the developed method demonstrate that the ABFD may be widely used for fundamental experiments in the field of mosquito physiology, for the development of new repellent chemicals and in evaluation studies of mosquito repellent products, such as anti-mosquito textiles. The further development of the membrane and feeding unit systems will enable a more practical evaluation of mosquito repellents and blood-feeding inhibitors, such as pyrethroids.
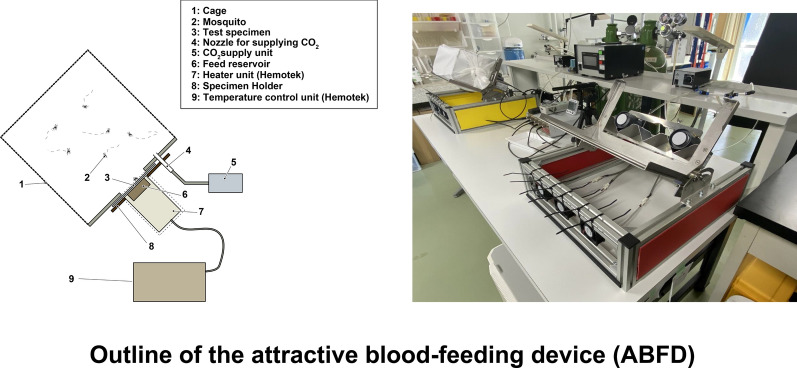

**Supplementary Information:**

The online version contains supplementary material available at 10.1186/s13071-021-04656-y.

## Background

The global threat of mosquito-borne infectious diseases is increasing because of the horizontal and vertical worldwide expansion of the vector mosquito species, in association with increasing human movement and global warming [1]. Accordingly, consumer needs for anti-mosquito textiles, with enhanced functionality for protecting against mosquito bites or limiting human exposure to mosquitoes, are increasing. The use of insecticide- or repellent-treated bed nets, curtains and other types of anti-mosquito products is also increasing. Over the years, long-lasting insecticidal nets (LLINs) and uniforms have played a major role in the control of mosquito-borne diseases [2]. The World Health Organization (WHO) has established guidelines for the laboratory and field testing of LLINs [3], which have similar properties as repellent-treated textiles and clothing. However, no international standard method exists for the evaluation of such mosquito repellent-treated products, although some country-specific methods have been published [4, 5]. The standardization of commercial textile product testing using an official testing method established by the International Organization of Standardization (ISO) is, therefore, indispensable for the authentic and objective evaluation of these products.

A regular supply of animal blood is essential for rearing and conducting experiments with blood-sucking insects in the laboratory. Although animal use is the most convenient means for rearing and experimental purposes, it requires ethical clearance, access to a dedicated animal breeding facility and alignment with environmental hygiene standards, which entails high maintenance costs. For example, great effort was required to obtain ethical clearance for a number of animal experiments recently reported [6–8]. Animal welfare concerns were originally addressed in the UK in 1959 by Russell and Burch [9], who proposed the 3R principles (*r*efinement, reduction of suffering; *r*eplacement, use of alternative methods; and *r*eduction, reduction of the number of animals per experiment); these have become the fundamental animal welfare principles in research. Artificial blood-feeding of insects that does not involve living animals has become a common means of mosquito rearing under policies based on these principles. Therefore, a new bioassay system for evaluating mosquito blood-feeding behavior that does not involve living animals is also indispensable.

The aim of the present study was to introduce a newly developed bioassay system with an artificial feeding device and to evaluate the blood-feeding responses and behavior of mosquitoes to some chemical repellents using this system. The results of this study can be used as a source of informative data for fundamental research, such as studies in mosquito physiology, the development of new repellent chemicals and the evaluation of mosquito repellent products, including anti-mosquito textiles.

## Methods

### Outline of the attractive blood-feeding device

**Fig. 1:**
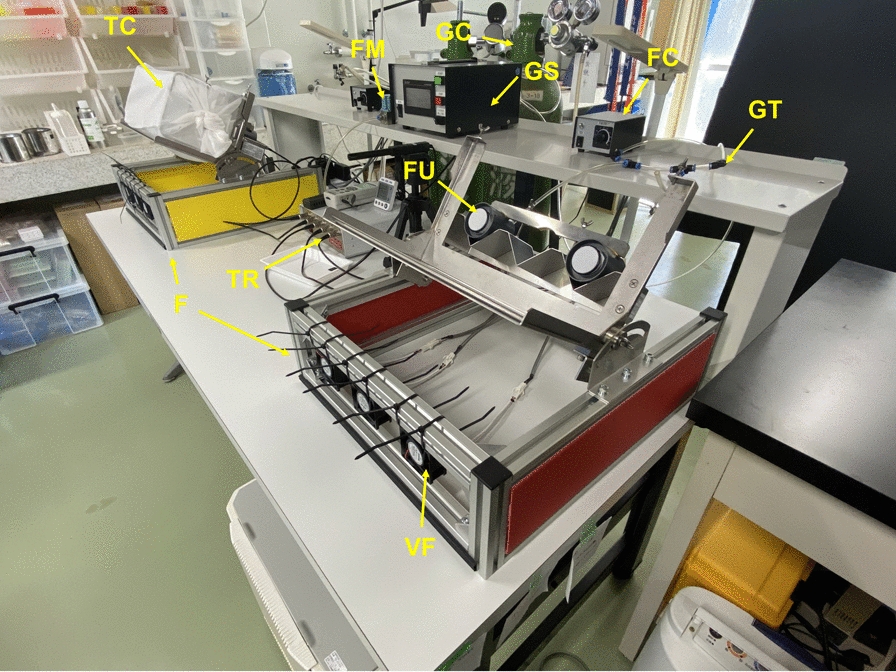
Overview of the attractive blood-feeding device (ABFD). The feeding unit (*FU*) consists of a feed reservoir (Hemotek Ltd., Blackburn, UK) with a polytetrafluoroethylene (PTFE) membrane and a heater unit (Hemotek Ltd). *F* Frame, *FC* ventilation fan controller, *FM* flow meter,* GC* carbon dioxide gas cylinder, *GS* carbon dioxide gas supply unit, *GT* carbon dioxide gas tubes, *TC* test cage, * TR* Thermostat temperature regulator (Hemotek Ltd.), *VF* ventilation fan

The design of the attractive blood-feeding device (ABFD) was inspired by a photoelectric sensing device for recording mosquito host-seeking behavior, reported in 2004 by Kawada and Takagi [10]. The original device used contrasting colors (black and white), intermittently discharged carbon dioxide and heating of the target as mosquito-attracting factors. The basic ABFD consists of a feeding unit, thermostat temperature regulator, sample holder, frame, carbon dioxide gas supply unit, ventilation fan and test cage (Fig. 1); additional components are test blood and a membrane.

#### Feeding unit

The feeding unit consists of a feed reservoir and a heater unit. The feed reservoir is composed of a metal or glass container holding the test blood and a membrane covering the container. The membrane-covered surface acts as a feeding surface. In the present study, a 3-ml standard reservoir (Hemotek Ltd.; product code OR37-25) was installed on the heater unit (Hemotek Ltd.; product code FU1).

#### Thermostat temperature regulator

The thermostat temperature regulator is connected to the feeding unit and maintains the feed reservoir at a constant temperature, with high accuracy. A PS5 power unit (Hemotek Ltd.; product code PS5B) was used in the present study to power the heater unit.

#### Sample holder

The sample holder is used for holding the test sample. It enables the quick mounting and removal of the sample from the test cage containing mosquitoes. It has a hole to expose the sample through which the attracted and resting mosquitoes can feed.

#### Frame

A metal frame was prepared for fastening the test cage and feeding unit. The frame fixes the feeding surface at a 45° angle from the horizontal plane. A maximum of three feeding units can be installed on the frame.

#### Carbon dioxide gas supply unit

The carbon dioxide gas supply unit consists of a carbon dioxide gas cylinder containing liquefied carbon dioxide, a solenoid valve controlled by a timer and a gas flow meter. In the present study, carbon dioxide gas was discharged through a nozzle at a flow rate of 1.0 l/min and at a constant time interval (10 s on and 50 s off) [10] onto the surface and in the vicinity of the test sample from the upper part of the feeding unit in the test cage.

#### Ventilation fan

To prevent carbon dioxide gas accumulating in the test cage, the setup was ventilated using three brushless DC fans (MISUMI Group Inc., Tokyo, Japan) located under the test cage. The wind speed was regulated at 1 m/s.

#### Test cage

**Fig. 2:**
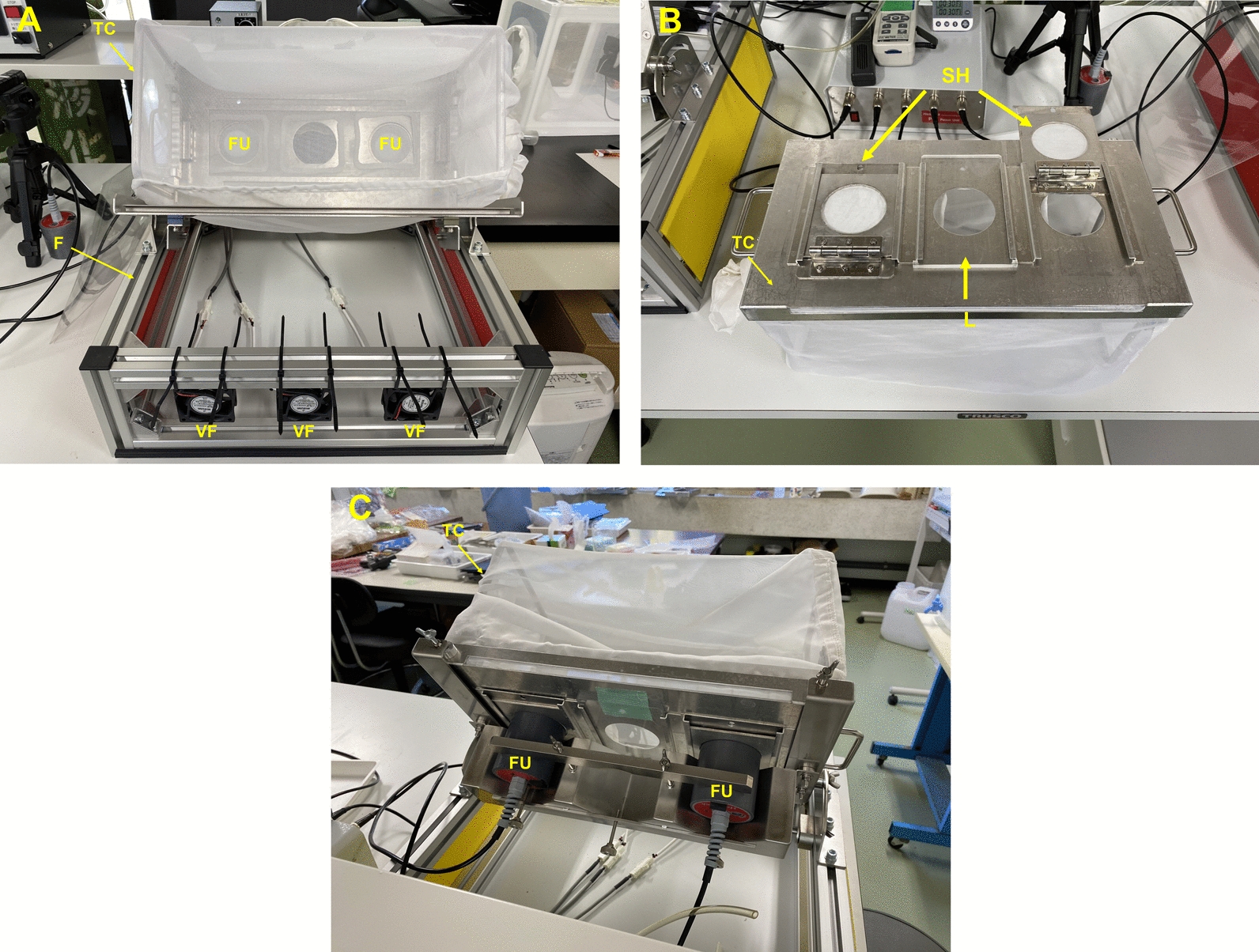
The ABFD test cage. **a** Test cage installed on the frame. **b** Bottom of the test cage. Two openings are covered with sample holders of binding cotton cloth and one opening (center) is closed with a lid. **c** Back view of the test cage. Feeding units are attached to the cotton cloth behind the test cage through openings in the sample holder.* L* Lid, *SH* sample holder; for other abbreviations, see Fig. 1 caption

The test cage is a cuboid (width 380 mm; height 200 mm; depth 200 mm), with three openings on the bottom that can be connected to the feeding units (Fig. 2a). The openings are joined to the feeding units* via* the sample holders (Fig. 2b, c). Three or two openings (without the center one) are used as required, according to the aim of the study. Mesh, fine enough to prevent the test mosquitoes from escaping, but not too tight to present observation of the test mosquitoes, was used for the other five faces of the test cage.

#### Test blood

Equine blood in Alsever’s solution (Japan Bio Serum, Tokyo, Japan; product code 003-00074) [11], prepared by mixing the blood with an anticoagulant (Alsever’s solution), was used in the present study. However, alternatives include a solution containing the required blood components or a similar solution that promotes mosquito blood-feeding.

#### Membrane

A polytetrafluoroethylene (PTFE)-based membrane is well suited to the study purpose [12]. We used Gaflon PTFE Thread Sealing Tape (3P Performance Plastics Products, Clichy, France; width 25.4 mm, thickness, 0.1 mm) in the present study. Other types of membranes that can be used include those made from animal intestine, animal skin, collagen film, sausage casing [11] or artificial skin.

#### Test mosquitoes

Unfed female adults of laboratory colonies of *Aedes albopictus* (Skuse) (Nagasaki and STS colonies) were used in the present study. An *Ae. albopictus* Nagasaki colony was collected in Nagasaki, Japan. An *Ae. albopictus* STS colony was supplied by Sumika Techno Service Corp. (Hyogo, Japan). Thirty mosquitoes were used per test, except when mentioned otherwise. The number of mosquitoes was determined according to the cage test method in WHO guidelines [3].

### Recommended test procedures for ABFD

**Fig. 3:**
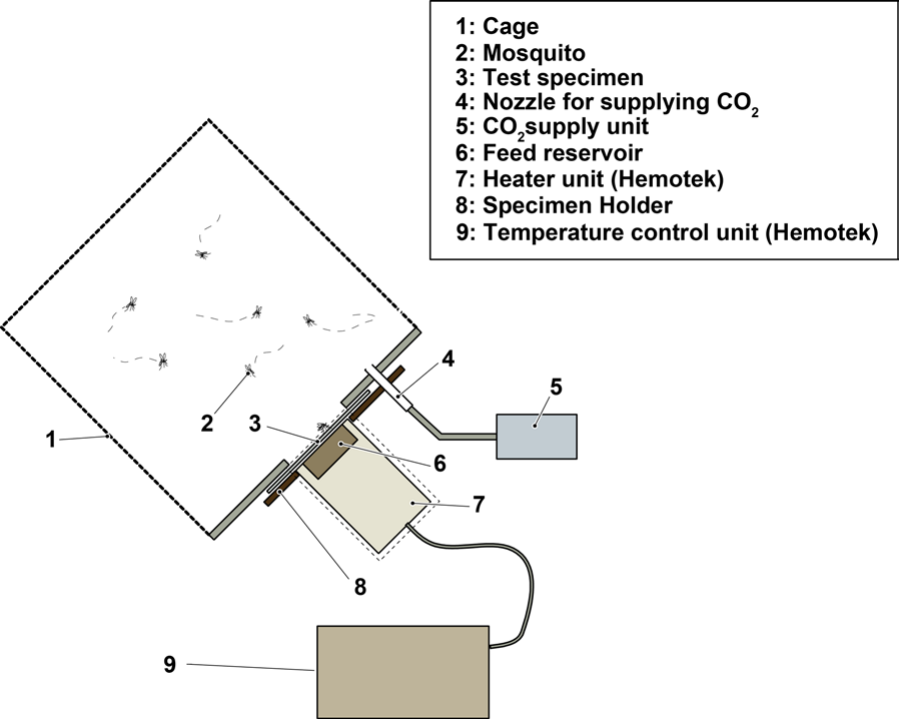
Diagram of the test cage connected with the feeding unit

Test chemicals were diluted with ethanol to the required concentration (% w/v). Ethanol solutions of the tested chemicals at the specified concentrations were then used to treat a 72-cm^2^ single-fiber cotton cloth (Japan Industry Standard test fabric JIS L0803) uniformly at 1 ml per 600 cm^2^, according to WHO guidelines [13], using an AutoRep™ E adjustable repeating dispenser (Mettler-Toledo Rainin, LLC, Oakland, CA, USA). After drying, the cloth was installed on the sample holder. Test mosquitoes were released into the test cage. To identify test mosquitoes with sufficient blood-feeding eagerness, mosquitoes exhibiting probing behavior on human hands held over the breeding cage were collected with an aspirator. Three milliliters of the test blood was poured into the feed reservoir and covered with the membrane. The feed reservoirs were installed on the heater unit until the temperature of the feeding surface reached 34 °C, as determined by an infrared surface thermometer. The test samples on the sample holders were placed at the openings of the test cage, and the cage and feeding units were then installed on the frame. The test samples were exposed to the inside of the cage by pushing on the feeding units (Fig. 3). Initiation of carbon dioxide gas release was as described above, and the number of mosquitoes resting on the feeding surface was recorded for 30 min at 1-min intervals, unless mentioned otherwise. After the final recording (30 min after the start of the experiment), the test samples and feeding units were removed (following the removal of the feeding units, the openings in the test cage were shut by lids). The test mosquitoes were killed by freezing, and the numbers of blood-fed and unfed mosquitoes were recorded. Testing was performed under laboratory conditions at 27 °C ± 2 °C, a relative humidity of between 50 and 85% and illuminance of at least 600 lx. The testing setup was such that the observer would not affect the test mosquitoes by exhalation, body temperature and movement.

### Experiment 1. ABFD performance validation

#### Comparative ABFD testing of mosquito attraction to feeding units with and without carbon dioxide discharge

Unfed females of an *Ae. albopictus* STS colony (10–20 days after emergence) were released into the test cage. Three feeding units were installed on the ABFD frame. A sausage casing made of swine intestine [11] was used as a membrane instead of the PTFE tape, and a knitted cotton cloth was used instead of the single-fiber cotton cloth. The numbers of mosquitoes attracted to and resting on the ABFD and blood-feeding mosquitoes were compared under two test conditions: with and without carbon dioxide discharge. The mosquitoes were observed for 15 min at 1-min intervals. The tests with and without carbon dioxide were done in 16 and nine replicates, respectively.

#### Comparative testing of mosquito attraction to the feeding unit and the human arm

Prior to the experiment, an *Ae. albopictus* STS colony was tested for the absence of potential infection with egg-borne transmitted viruses. Viral RNA was extracted using TRIzol-LS reagent (Thermo Fisher Scientific K.K., Tokyo, Japan), and a conventional reverse-transcription PCR was performed using primers specific for flaviviruses [14], common Dengue virus [15], Toga–alphaviruses [16] and Rift Valley fever virus [17].

For the comparative experiment, 20 unfed females of the *Ae. albopictus* STS colony (10–20 days since emergence) were released into the test cage. Sausage casing made of swine intestine was used as a membrane instead of the PTFE tape and a knitted cotton cloth was used instead of the single-fiber cotton cloth. For testing of the feeding units, two feeding units with untreated cotton cloths were installed on the ABFD frame. For testing of human subjects as an attractant, volunteers exposed a portion of their arm through the two openings of the test cage to equalize the exposed attractant area in both tests. Three male volunteers, one each in their forties (A), fifties (B) and sixties (C), respectively, and one female in her thirties (D) participated in the test. The numbers of attracted and resting mosquitoes and blood-feeding mosquitoes were compared under the two conditions. The observations were made for 10 min, at 0.5-min intervals for the first 6 min and then at 1-min intervals for the remaining 4 min. Testing involving the feeding units was repeated five times and that involving human subjects was repeated six times (1 time each with volunteers A, C, and D, and 3 repeats with volunteer B).

### Experiment 2. Comparative ABFD testing of chemical repellent activity against mosquitoes

The performance of the test chemicals to repel mosquito and their effect on mosquito behavior were assessed using the ABFD. Two different tests (choice test and no-choice test) were performed using an *Ae. albopictus* Nagasaki colony, and *N*,*N*-diethyl-*m*-toluamide (DEET), 2-(2-hydroxyethyl)-1-piperidinecarboxylic acid 1-methylpropyl ester (icaridin) and 3-phenoxybenzyl (1RS)-*cis,trans*-3-(2,2-dichlorovinyl)-2,2-dimethylcyclopropanecarboxylate (permethrin).

#### Choice test

Two feeding units with a single-fiber cotton cloth, a chemically treated cloth and untreated cloth, respectively, were used in the test. Ethanol solutions of the chemicals were prepared at different concentrations (%, w/v) and then used to treat the surface of the cotton cloth at 1 ml per 600 cm^2^, using an AutoRep E Repeating Dispenser (Mettler-Toledo Rainin, LLC). The control cotton cloth was treated with the same amount of ethanol. After drying for 15 min, the cloths were installed on the feeding units. Mosquitoes attracted to and resting on the treated (*N*_t_) and untreated cloths (*N*_c_) were counted, and the overall blood-feeding rates, irrespective of whether the mosquitoes fed on the treated or untreated cloths, were recorded. Percentage repellency was calculated as [1 − (*N*_t_/*N*_c_)] × 100. Blood-feeding rates were calculated as the percentage of the number of blood-fed mosquitoes per total number of mosquitoes tested (30 mosquitoes per replicate). Three replicate experiments were performed per concentration tested.

#### No-choice test

Two feeding units, each with a cotton cloth treated equally with the same chemical, were used in the test. Mosquitoes attracted to and resting on the treated cloths (*N*′_t_) were counted and the blood-feeding rates recorded. A control test (both sides of feeding units were untreated) was conducted once at the beginning of the test day using the same mosquito colony. The repellency of the chemicals was evaluated by comparing the numbers of mosquitoes recorded when the repellent-treated clothes were used to the numbers recorded when two untreated cloths were used (*N*′_c_). Percentage repellency was calculated as [1 − (*N*′_t_/*N*′_c_)] × 100. Blood-feeding rates were calculated as the percentage of the number of blood-fed mosquitoes per total number of mosquitoes tested (30 mosquitoes per replicate). Three replicate experiments were performed per concentration tested.

### Experiment 3. Observation of the behavior of mosquitoes exposed to DEET, icaridin and permethrin

A feeding unit of the ABFD was used for this observation. Unfed females of *Ae. albopictus* (Nagasaki colony) were released into a transparent plastic case (7 × 7 × 10 cm), the top and bottom sides of which had a window (diameter 4 cm) made of fine mesh. A feeding unit covered by a chemically treated cotton gauze was attached at the bottom side of the case, allowing the mosquitoes to probe the surface of the feeding unit. A small brushless DC fan was installed at the top for ventilation. Mosquito behavior on or in the vicinity of the surface of the feeding unit treated with repellents (DEET and icaridin) or pyrethroid (permethrin) was observed using the video function of a single-lens reflex camera (EOS M5; Canon Inc., Tokyo, Japan) or a high-speed camera (Fastcam Mini AX; Photoron Ltd., Tokyo, Japan).

### Data analysis

The attraction of mosquitoes and blood-feeding were analyzed using a Wilcoxon signed-ranks test or Kruskal–Wallis one-way analysis of variance, and Dunn’s multiple comparisons test using JMP Pro 15.0.0 (SAS Institute Japan Inc. Tokyo, Japan).

## Results

### Experiment 1

#### Comparative attractiveness of the ABFD with and without carbon dioxide discharge to *Ae. albopictus*

The intermittent discharge of carbon dioxide significantly increased the number of mosquitoes (*Ae. albopictus* STS colony) attracted to and resting on the feeding units (*P* < 0.0001,* df* = 1, *χ*^2^ = 136). The average number of mosquitoes attracted to and resting on the feeding units per 1 min with and without carbon dioxide discharge was 9.37 (95% confidence interval [CI] 8.82–9.92) and 4.21 (95% CI 3.79–4.64), respectively (Additional file 1: Figure S1). The blood-feeding rate was also significantly increased with carbon dioxide discharge (*P* = 0.0003,* df* = 1, *χ*^2^ = 13.3) (Additional file 2: Figure S2). The average percentage of blood-fed mosquitoes was 52.2 and 21.4% with and without carbon dioxide, respectively.

#### Comparison of attractivness of the feeding unit and human arm to mosquitoes

As determined by reverse-transcription PCR, no mosquitoes from the STS colony were infected by flaviviruses, common Dengue virus, Toga-alphaviruses and Rift Valley fever virus, which confirmed that these mosquitoes were safe to use in tests involving human volunteers. The average numbers of mosquitoes attracted to and resting on the feeding unit and the human skin per 10 min were 6.50 (95% CI 5.98–7.18) and 0.81 (95% CI 0.62–1.00), respectively; this difference was statistically significant (*P* < 0.0001,* df* = 1 *χ*^2^ = 117) (Additional file 3: Figure S3). The average blood-feeding rate was 23% for the feeding unit and 3.4% for human skin; this difference was statistically significant (*P* = 0.0071,* df* = 1, *χ*^2^ = 7.24) (Additional file 4: Figure S4).

### Experiment 2

#### Repellency of DEET, icaridin and permethrin against* Ae. albopictus* as determined by the ABFD choice test

For 0.1% DEET, the average numbers of mosquitoes attracted to and resting on the treated and untreated feeding unit per 1 min were 0.70 (95% CI 0.45–0.95) and 0.92 (95% CI 0.70–1.15), respectively; for 1% DEET, these were 0.044 (95% CI 0.0010–0.088) and 0.94 (95% CI 0.74–1.15), respectively; and for 2% DEET, they were 0 and 0.21 (95% CI 0.13–0.30), respectively. Significant differences in the numbers of mosquitoes attracted to and resting on the DEET-treated and untreated feeding units were observed for all concentrations tested: 0.1% DEET (*P *= 0.026,* df* = 1, *χ*^2^ = 4.94), 1% DEET (*P *< 0.0001,* df* = 1, *χ*^2^= 66.9) and 2% DEET (*P* < 0.001,* df* = 1, *χ*^2^ = 21.1). Percentage repellency values for 0.1, 1 and 2% DEET were 23.9, 95.3 and 100%, respectively (Table 1).

**Table 1:** Repellency of chemicals against *Aedes albopictus*, as determined by the choice test

Chemical	Concentration of chemical tested (%)	Inhibition of resting (%)	Inhibition of blood-feeding (%)
DEET	0.1	23.9	57.8
	1	95.3	82.2
	2	100	100
Icaridin	0.1	46.0	87.8
	1	65.5	90.0
	2	100	98.9
Permethrin	2	31.6	81.1

DEET: *N*,*N*-Diethyl-*m*-toluamide

The average numbers of mosquitoes attracted to and resting on the icaridin-treated and untreated feeding units per 1 min were 0.54 (95% CI 0.35–0.74) and 1.0 (95% CI 0.68–1.32) for 0.1% icaridin, respectively; 0.49 (95% CI 0.35–0.63) and 1.42 (95% CI 1.11–1.72) for 1% icaridin, respectively; and 0.011 (95% CI − 0.011 to 0.033) and 1.64 (1.19–2.10) for 2% icaridin, respectively. Significant differences in the number of mosquitoes attracted to and resting on the treated and untreated feeding units were observed for 1% (*P* < 0.0001,* df* = 1, *χ*^2^ = 25.9) and 2% icaridin (*P* < 0.0001,* df*=1, *χ*^2^ = 47.7), whereas the difference was not significant for 0.1% icaridin (*P* = 0.17,* df* = 1, *χ*^2^ = 1.86). Percentage repellency values for 0.1, 1 and 2% icaridin were 46.0, 65.5 and 99.3%, respectively (Table 1).

**Fig. 4:**
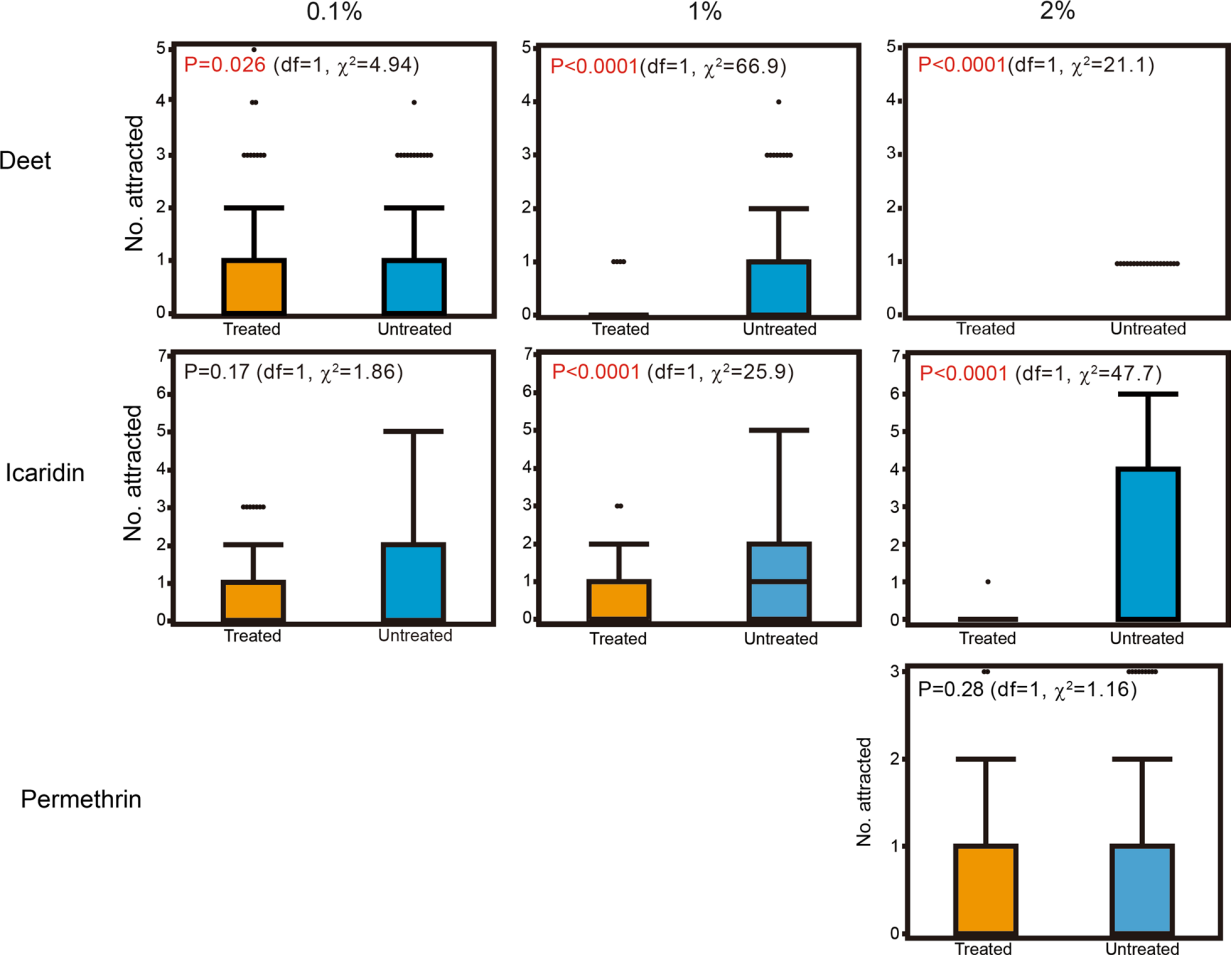
Boxplot of number of unfed *Aedes albopictus* females attracted to and resting on the surface of the feeding unit, treated or untreated with chemicals in the choice test. Letters in the graphs indicate the calculated probability (Kruskal–Wallis one-way analysis of variance)

For 2% permethrin, the average numbers of mosquitoes attracted to and resting on the treated and untreated feeding units per 1 min were 0.54 (95% CI 0.40–0.69) and 0.79 (95% CI 0.58–1.00), respectively; the difference was not significant (*P *= 0.28,* df* = 1, *χ*^2^ = 1.16). Percentage repellency for 2% permethrin was 31.6% (Fig. 4; Table 1). No knockdown of mosquitoes was observed during permethrin testing.

**Fig. 5:**
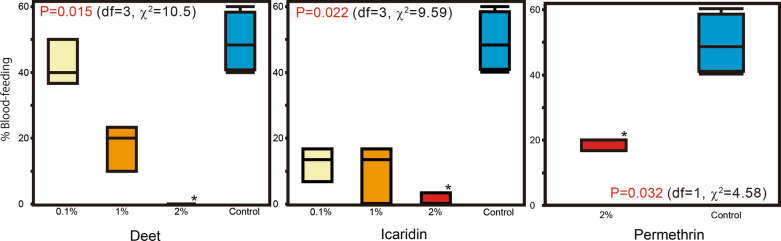
Boxplot of blood-feeding rates of unfed *Ae. albopictus* females in the choice test. Letters in the graphs indicate the calculated probability (Kruskal–Wallis one-way analysis of variance). Asterisks indicate significant difference from the control (Dunn’s multiple comparison test)

The average blood-feeding rates in the choice test were 42.2, 17.8 and 0% for 0.1, 1 and 2% DEET treatments, respectively; 12.2, 10.0 and 1.1% for the 0.1, 1 and 2% caridin treatments, respectively; and 18.9% for the 2% permethrin treatment. The average blood-feeding rate for the control test (both feeding units untreated) was 49.2%. Differences among the concentrations were significant for all chemicals tested: DEET (*P *= 0.015,* df* = 3, *χ*^2^ = 10.5), icaridin (*P* = 0.022,* df* = 3, *χ*^2^ = 9.59) and permethrin (*P*=0.032,* df* = 1, *χ*^2^= 4.58). Significant differences from the control blood-feeding rate were observed for 2% DEET (*P* = 0.022), 2% icaridin (*P* = 0.023) and 2% permethrin (*P* = 0.050) (Fig. 5; Table 1).

### Repellency of DEET, icaridin, and permethrin against *Ae. albopictus* as determined by the ABFD no-choice test

Differences among the concentrations were significant for all chemicals tested: DEET (*P *< 0.0001,* df* = 3, *χ*^2^ = 182), icaridin (*P*<0.0001,* df* = 4, *χ*^2^=221) and permethrin (*P* < 0.0001,* df* = 1, *χ*^2^ = 60.2). The average numbers of mosquitoes attracted to and resting on the treated feeding unit per 1 min were 1.17 (95% CI 0.84–1.50), 0.089 (95% CI 0.029–0.15) and 0.089 (95% CI 0.0021–0.18) for the 0.1, 1 and 2% DEET treatments, respectively. There were significant differences between the number of attracted and resting mosquitoes in the control feeding unit (2.27 [95% CI 1.91–2.63] and the number in each DEET treatment (*P* < 0.0001). Percentage repellency values for 0.1, 1 and 2% DEET were 48.5, 96.1 and 96.1%, respectively.

The average numbers of mosquitoes attracted to and resting on the treated feeding unit per 1 min were 1.31 (95% CI 1.10–1.52), 0.69 (95% CI 0.58–0.80), 0.28 (0.15–0.41) and 0.011 (95% CI − 0.011 to 0.033) for the 0.1, 1, 2 and 4% icaridin treatments, respectively. There were significant differences between the number of attracted and resting mosquitoes in the control test and the numbers observed for the 1, 2 and 4% icaridin treatments (*P* < 0.0001); the difference between the control and 0.1% icaridin was not significant (*P *= 0.21). Percentage repellency values for 0.1, 1, 2 and 4% icaridin were 42.3, 69.6, 87.7 and 99.5%, respectively.

**Fig. 6:**
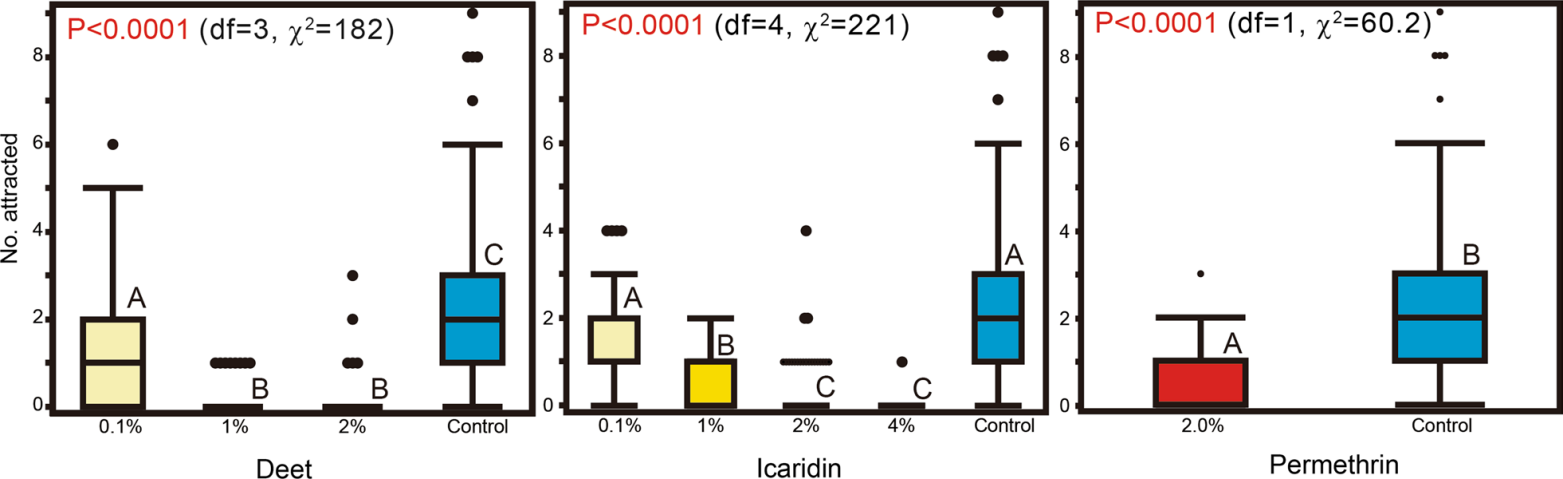
Boxplot of number of unfed *Ae. albopictus* females attracted to and resting on the surface of the feeding unit treated with chemicals in the no-choice test. Letters in the graphs indicate calculated probability (Kruskal–Wallis one-way analysis of variance). Different capital alphabetical letters in the graph indicate significant differences in numbers among the concentrations (Dunn’s multiple comparison test)

For 2% permethrin, the average number of attracted and resting mosquitoes was 0.53 (0.40–0.67). The difference from the number of mosquitoes attracted and resting in the control test was significant (*P* < 0.0001). Percentage repellency % for 2% permethrin was 76.7% (Fig. 6; Table 2). No knockdown of mosquitoes was observed during permethrin testing.

**Table 2:** Repellency of chemicals against *Ae. albopictus*, as determined by the no-choice test

Chemicals	Concentration (%)	Inhibition of resting (%)	Inhibition of blood-feeding (%)
DEET	0.1	48.5	74.4
	1	96.1	90.0
	2	96.1	100
Icaridin	0.1	42.3	53.3
	1	69.6	80.0
	2	87.7	80.0
	4	99.5	100
Permethrin	2	76.7	95.6

**Fig. 7:**
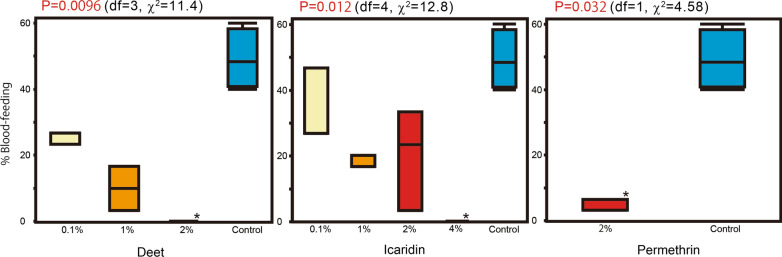
Boxplot of blood-feeding rates of unfed *Ae. albopictus* females in the no-choice test. Letters in the graphs indicate calculated probability (Kruskal–Wallis one-way analysis of variance). Asterisks indicate significant differences from the control (Dunn’s multiple comparison test)

The average blood-feeding rates in the no-choice test were 25.6, 10.0 and 0% for the 0.1, 1, and 2% DEET treatments, respectively; 33.3 17.8, 20.0 and 0% for the 0.1, 1, 2 and 4% icaridin treatments, respectively; and 4.4% for the 2% permethrin treatment. Differences among the concentrations were significant for all chemicals tested: DEET (*P *= 0.0096,* df* = 3, *χ*^2^=11.4), icaridin (*P* = 0.012,* df* = 4, *χ*^2^ = 12.8) and permethrin (*P* = 0.032,* df* = 1, *χ*^2^=4.58). Significant differences from the control blood-feeding rate (49.2%) were observed for 2% DEET (*P *= 0.011), 4% icaridin (*P *= 0.012) and 2% permethrin (*P *= 0.050) (Fig. 7, Table 2).

### Experiment 3

#### Behavior of mosquitoes exposed to DEET, icaridin and permethrin

Mosquitoes on the chemical-untreated surface exhibited frequent probing behavior, by walking on the surface (Additional file 5: Video S1) after touching down on it after flight (Additional file 6: Video S2). Almost no physical mosquito contact was observed on a 10% DEET-treated surface (Additional file 7: Video S3). Avoidance flight without touching the DEET-treated surface was revealed by a high-speed video (Additional file 8: Video S4). Some mosquitoes were found on the 10% icaridin-treated surface; however, they did not show probing behavior, in contrast to what was observed on the untreated surface (Additional file 9: Video S5). On the permethrin-treated surface, mosquitoes were observed grooming the antennae with forelegs and walking on the surface without probing, and then escaping the permethrin-treated area (Additional file 10: Video S6).

## Discussion

The increasing worldwide threat of mosquito-borne infectious diseases is fueling interest in repellent-treated materials and their testing. Many studies of mosquito host-seeking behavior involve field and laboratory tests relying on the collection and counting of mosquitoes that land on human or animal baits. Such studies, however, are labor intensive, costly, time consuming and involve the risk of disease transmission to human volunteers. Here, we present the ABFD, an accurate simulated laboratory setup that allows the extrapolation of laboratory findings to the field. It constitutes a possible breakthrough in the study of mosquito behavior.

Previous studies of mosquito attractants have identified several key factors that attract host-seeking female mosquitoes, including carbon dioxide, 1-octen-3-ol, l-lactic acid, heat, color and light [18–20]. These studies led to the development of devices that enable automatic recording of mosquito behavior in the laboratory [10, 21, 22]. Among the mosquito attractant cues, carbon dioxide and heat are thought to be most important. In the present study, the intermittent discharge of carbon dioxide [10, 23] greatly enhanced not only mosquito attraction to the blood-feeding target but also the blood-feeding rate. Carbon dioxide, in combination with an additional attractant cue, such as color, odor and heat, might enhance the blood-feeding activity of mosquitoes as well as host-seeking eagerness [24, 25]. Carbon dioxide also increases repeated resting on heat target [26].

In the present study, mosquito attraction to and blood-feeding rate on the ABFD exceeded those to/on human skin. Here, the exposure area of human skin was the same as that of the feeding unit (approx. 4-cm in diameter), for comparable testing conditions. Furthermore, the amount of carbon dioxide discharged in the ABFD was approximately the same as that discharged by human breathing. The carbon dioxide concentration might be lower in the ABFD than around humans, as carbon dioxide was intermittently discharged only for 10 s per 1 min under continuous ventilation, whereas human breaths were discharged continuously under no ventilation. According to a recent study, *Aedes aegypti* uses local cues, such as heat plume, moisture and skin volatiles, in the vicinity of a human host [27]. Stimulation of mosquitoes with such local cues in the ABFD might be stronger than that by the human skin with a limited exposure area.

The most popular synthetic repellents currently in use are DEET, icaridin, and IR3535 (ethyl butylacetylaminopropionate). Repellents of natural origin, such as lemon, eucalyptus, and citronella are also widely used. Despite its relative toxicity [28], DEET is the reference repellent of the WHO [13] because it is the most efficient and long-lasting repellent available [29]. The recommended concentration of DEET ranges from 7 to 10% for a short-term effective duration (< 2 h), and from 20 to 30% for longer periods (up to 6 h) [29]. Icaridin is a newly developed mosquito repellent [30]. The recommended concentration of icaridin is 5–10% for short-term protection (3–5 h), and 20% for longer periods (up to 10 h) [29]. Permethrin, a well-known pyrethroid insecticide, has a long insecticidal history [13]. Its use as a repellent was first suggested in the 1970s, when it was used for the treatment of military clothing [31], and it was later registered by the US Environmental Protection Agency (in 1990) as a repellent for military clothing. Unlike other insect repellents that can be topically applied to human skin, permethrin is applied to clothing and outdoor-gear material [29]. It is a relatively safe insecticide, but its use for the treatment of clothes should be investigated in more detail [32].

In the present study, the choice test was devised to allow the mosquitoes to select the untreated attractive target if repelled by the chemical-treated target. The experiment revealed that DEET was sufficiently effective at a concentration of > 1%, whereas  a concentration of > 2% icaridin was required for similar effectivity. The effect of permethrin in this test was unique. Mosquitoes selected both attractants in the 2% treatment (Table 3). As revealed by video analyses, mosquitoes contacted and stayed on the permethrin-treated surface, although the orientation time was shortened by the irritant effect of permethrin. The same behavior was noted in a different test of permethrin using the WHO cone [33]. Also, a significant increase in the frequency of takeoffs from the permethrin-treated surface and in flying times, compared with those from the untreated surface, has been observed in *Anopheles gambiae* Giles, *An. arabiensis* Patton and *An. funestus* Giles [34].

**Table 3:** Concentration of chemicals required for specific inhibition of resting and blood-feeding behavior of *Ae. albopictus*

Chemical	Concentration required for 95% inhibition of resting (%)		Concentration required for 100% inhibition of blood-feeding (%)	
	Choice test	No-choice test	Choice test	No-choice test
DEET	0.1–1	0.1–1	1–2	1–2
Icaridin	1–2	2–4	> 2	2–4
Permethrin	> 2	> 2	> 2	> 2

Of note, in the present study, the number of mosquitoes attracted to and resting on the untreated feeding unit was significantly reduced in the choice test with 2% DEET, whereas no such reduction was observed with icaridin, permethrin and lower doses of DEET. The blood-feeding rate was also remarkably reduced in the 2% DEET treatment. In the choice test, the numbers of attracted and resting mosquitoes and the blood-feeding rates were a sum of those attracted and resting and feeding on the treated and untreated feeding units, as the mosquitoes could freely approach both the treated and untreated targets. This indicates that DEET treatment of the feeding unit affected the mosquitoes, even in the absence of direct contact with the treated surface. A similar reduction in the blood-feeding rate was observed for 2% icaridin, but the effect was more pronounced for DEET (Table 3). As revealed by the video footage, mosquitoes were frequently observed contacting the icaridin-treated surface, whereas the frequency of contact was very low on the DEET-treated surface. The differences in the modes of DEET and icaridin activity are mainly associated with the difference in the vapor pressure of the two chemicals. The vapor pressure of DEET (5.6 × 10^–3^ mmHg at 20 °C; https://www.atsdr.cdc.gov/ToxProfiles/tp185-c4.pdf) is more than 12-fold higher than that of icaridin (4.43 × 10^–4^ mmHg at 25 °C) [35]. In one study, the irritancy (repellency) of DEET (2%) against *Ae. aegypti* (L.) was significant up to 40 mm from the DEET-treated target, with no significant irritancy observed for an icaridin (6%)-treated surface even 2 mm from the treated target, indicating that irritancy (repellency) was only caused by tarsal contact with icaridin [36].

The dose-dependent reaction to repellents was more remarkable in the no-choice tests than in the choice tests. High repellency was observed for 1 and 2% DEET, 2 and 4% icaridin and 2% permethrin, although the percentage repellency for permethrin was lower (76.7%) than that for equivalent concentrations of DEET and icaridin. The blood-feeding rates indicated the similar effectiveness of 2% DEET, 4% icaridin and 2% permethrin (Table 2).

The number of studies on the comparative efficacy of DEET and icaridin are limited, and interpretation of the results is not unambiguous. These studies indicate little difference between the efficacy of DEET and icaridin, except for some evidence pointing to a higher persistence of icaridin on human skin [37]. In addition, most of the abovementioned studies involve human volunteers in the field or under laboratory conditions, with many possible sources of bias, such as that associated with individual (human) variation, climate, environment and mosquito species. In the present study, testing using the ABFD allowed a precise comparison of repellent chemicals without the abovementioned biases and ethical issues.

Testing methods for conventional mosquito repellents directly applied onto human skin are applicable to the testing of textiles treated with those materials and* vice versa*. Although pyrethroids are not recommended for the direct treatment of human skin because of dermal toxicity [38], they are used as contact repellents in treated textiles. The evaluation of the excito-repellency of pyrethroids, therefore, should be incorporated into the testing methods. The most frequently used laboratory testing methods for repellents are the cage, cone and excito-chamber tests. The cage test is the most common and simple of these [13]; however, ethical consent from volunteers has to be obtained prior to testing and, preferably, only pathogen-free mosquitoes tested. Furthermore, individual variations among volunteers (i.e. sex and age) may lead to data discrepancies. The cone and excito-repellency chamber tests [33, 34, 39] are ethically acceptable methods because they do not involve human volunteers or animals as mosquito attractants. Olfactometer and choice test systems are also recommended for precise evaluations [40]. A simulated blood-feeding test using heated defibrinated animal (swine or chicken) blood and Parafilm as a membrane, prescribed by the Guobiao (GB) national standard in China (GB/T 30126-2013), is a testing method that is most similar to the ABFD approach [4], although the attractiveness of defibrinated animal blood is inferior to that of the blood in Alsever’s solution, and Parafilm is not as good a membrane as PTFE and animal intestine [11]. The testing method using the ABFD was first developed for the evaluation of anti-mosquito textiles in 2018 [5]. As shown in the present study, its accuracy and reproducibility indicate that the ABFD can be widely used for fundamental research, such as in studies on mosquito physiology, the development of new repellent chemicals and the evaluation of mosquito repellent products, including anti-mosquito textiles.

## Conclusions

The results of this study show that the ABFD can be used to evaluate the biological performance of chemicals that show repellency to mosquitoes. However, the overall performance of the repellents should be evaluated also taking into consideration their volatility, skin permeability and degradation in the environment. The development of a membrane system that simulates the human skin and a novel feeding unit system that allows the long-term stability of blood, without reducing its attractiveness by coagulation and desiccation, will help address the above issues.

## Supplementary Information

**Additional file 1**: **Figure S1**. Effect of intermittently discharged carbon dioxide (10 s ON/50 s OFF, at the rate of 1.0 l/min) on the attraction of unfed* Ae. albopictus *females

**Additional file 2**: **Figure S2**. Effect of intermittently discharged carbon dioxide on the blood-feeding rate of unfed* Ae. albopictus* females

**Additional file 3**: **Figure S3**. Difference in the attractiveness of the feeding units and human arm to unfed* Ae. albopictus* females under the same conditions using the test cage of ABFD

**Additional file 4**: **Figure S4**. Difference in the blood-feeding rates of the feeding units and the human skin to unfed* Ae. albopictus* females under the same conditions using the test cage of ABFD

**Additional file 5**: **Video S1**. Mosquito behavior on an untreated surface (PPTX 17854 KB)

**Additional file 6**: **Video S2**. Mosquito behavior on an untreated surface, registered by a high-speed camera

**Additional file 7**: **Video S3**. Mosquito behavior on a surface treated with 10% DEET

**Additional file 8**: **Video S4**. Mosquito behavior on a surface treated with 10% DEET, registered by a high-speed camera

**Additional file 9**: **Video S5**. Mosquito behavior on a surface treated with 10% icaridin

**Additional file 10**: **Video S6**. Mosquito behavior on a surface treated with 10% permethrin

## Data Availability

The datasets used and/or analyzed during the current study are available from the corresponding author on reasonable request.

## Abbreviations

ABFD: Attractive blood-feeding device
DEET: *N*,*N*-Diethyl-*m*-toluamide
LLIN: Long-lasting insecticidal net
PTFE: Polytetrafluoroethylene
WHO: World Health Organization

## References

[1] Kawada H, Futami K, Higa Y, Rai G, Suzuki T, Rai SK (2020). Distribution and pyrethroid resistance status of *Aedes aegypti* and *Aedes albopictus* populations and possible phylogenetic reasons for the recent invasion of *Aedes aegypti* in Nepal. Parasites Vectors.

[2] Carnevale P, Gay F, Ariey F, Gay F, Ménard R (2019). Insecticide-treated mosquito nets. Malaria control and elimination. Methods in molecular biology.

[3] World Health Organization. Guidelines for laboratory and field testing of long-lasting insecticidal mosquito nets. 2005. https://apps.who.int/iris/bitstream/handle/10665/69007/WHO_CDS_WHOPES_GCDPP_2005.11.pdf. Accessed 2 March 2021.

[4] Guobiao (GB; National Standard in China). Textiles—testing and evaluation for anti-mosquitoes properties (in Chinese). 2014. http://www.texfunction.com/view746.html. Accessed 2 March 2021.

[5] Japanese Industrial Standard. JIS L 1950–1:2018 Textile–anti-mosquito performance test method—Part 1: test method for the attractive blood-feeding apparatus. 2018. https://webdesk.jsa.or.jp/preview/pre_jis_l_01950_001_000_2018_e_ed10_i4.pdf. Accessed 2 March 2021.

[6] Laidoudi Y, Tahir D, Medkour H, Varloud M, Mediannikov O, Davoust B (2020). Effect of dinotefuran, permethrin, and pyriproxyfen (Vectra® 3D) on the foraging and blood-feeding behaviors of *Aedes albopictus* using laboratory rodent model. Insects.

[7] Schreur PJW, Vloet RPM, Kant J, Van Keulen L, Gonzales JL, Visser TM (2021). Reproducing the Rift Valley fever virus mosquito-lamb-mosquito transmission cycle. Sci Rep..

[8] Muhammad NAF, Kassim NFA, Majid AHA, Rahman AA, Dieng H, Avicor SW (2020). Biting rhythm and demographic attributes of *Aedes albopictus* (Skuse) females from different urbanized settings in Penang Island, Malaysia under uncontrolled laboratory conditions. PLOS ONE.

[9] Russell WMS, Burch RL (1959). The principles of humane experimental technique.

[10] Kawada H, Takagi M (2004). Photoelectric sensing device for recording mosquito host-seeking behavior in the laboratory. J Med Entomol.

[11] Tsurukawa C, Kawada H (2014). Experiment on mosquito blood feeding using the artificial feeding device. Med Entomol Zool.

[12] Siria DJ, Batista EPA, Opiyo MA, Melo EF, Sumaye RD, Ngowo HS (2018). Evaluation of a simple polytetrafluoroethylene (PTFE)-based membrane for blood-feeding of malaria and dengue fever vectors in the laboratory. Parasites Vectors.

[13] World Health Organization. Guidelines for efficacy testing of mosquito repellents for human skin. 2009. https://apps.who.int/iris/bitstream/handle/10665/70072/WHO_HTM_NTD_WHOPES_2009.4_eng.pdf?sequence=1. Accessed on 3 March 2021.

[14] Tanaka M (1993). Rapid identification of flavivirus using the polymerase chain reaction. J Virol Method.

[15] Lanciotti RS, Calisher CH, Gubler DJ, Chang GJ, Vorndam AV (1992). Rapid detection and typing of Dengue viruses from clinical samples by using reverse transcriptase-polymerase chain reaction. J Clin Microbiol.

[16] Pfeffer M, Proebster B, Kinney RM, Kaaden OR (1997). Genus-specific detection of alphaviruses by a semi-nested reverse transcription-polymerase chain reaction. Am J Trop Med Hyg.

[17] Njenga MK, Paweska J, Wanjala R, Rao CY, Weiner M, Omballa V (2009). Using a field quantitative real-time PCR test to rapidly identify highly viremic Rift Valley fever cases. J Clin Microbiol.

[18] Takken W, Kline DL (1989). Carbon dioxide and 1-octen-3-ol as mosquito attractants. J Am Mosq Control Assoc.

[19] Kline DL, Wood JR, Cornell JA (1991). Interactive effects of 1-octen-3-ol and carbon dioxide on mosquito (Diptera: Culicidae) surveillance and control. J Med Entomol.

[20] Pates HV, Takken W, Stuke K, Curtis CF (2001). Differential behaviour of *Anopheles gambiae* sensu stricto (Diptera: Culicidae) to human and cow odours in the laboratory. Bull Entomol Res.

[21] Chiba Y, Yamakado C, Kubota M (1981). Circadian activity of the mosquito *Culex pipiens molestus* in comparison with its subspecies *Culex pipiens pallens*. Int J Chronobiol.

[22] Yee WL, Foster WA (1992). Diel sugar-feeding and host-seeking rhythms in mosquitoes (Diptera: Culicidae) under laboratory conditions. J Med Entomol.

[23] Maekawa E, Aonuma H, Nelson B, Yoshimura A, Tokunaga F, Fukumoto S (2011). The role of proboscis of the malaria vector mosquito *Anopheles stephensi* in host-seeking behavior. Parasites Vectors.

[24] Zhou YH, Zhang ZW, Fu YF, Zhang GC, Yuan S (2018). Carbon dioxide, odorants, heat and visible cues affect wild mosquito landing in open spaces. Front Behav Neurosci.

[25] McMeniman CJ, Corfas RA, Matthews BJ, Ritchie SA, Vosshall LB (2014). Multimodal integration of carbon dioxide and other sensory cues drives mosquito attraction to humans. Cell.

[26] Liu MZ, Vosshall LB (2019). General visual and contingent thermal cues interact to elicit attraction in female *Aedes aegypti* mosquitoes. Curr Biol.

[27] van Breugel F, Riffell J, Fairhall A, Dickinson MH (2015). Mosquitoes use vision to associate odor plumes with thermal targets. Curr Biol.

[28] Nguyen QD, Vu MN, Hebert AA (2018). Insect repellents: An updated review for the clinician. J Am Acad Dermatol..

[29] Tavares M, da Silva MRM, de Siqueira LBO, Rodrigues RS, Bodjolle-d'Almeida L, dos Santos EP (2018). Trends in insect repellent formulations: a review. Int J Pharm.

[30] Paumgartten FJR, Delgado IF (2016). Mosquito repellents, effectiveness in preventing diseases and safety during pregnancy. Vigil Sanit Debate.

[31] Schreck CE, Posey K, Smith D (1978). Durability of permethrin as a potential clothing treatment to protect against blood-feeding arthropods. J Econ Entomol.

[32] Appel KE, Gundert-Remy U, Fischer H, Faulde M, Mross KG, Letzel S (2008). Risk assessment of Bundeswehr (German Federal Armed Forces) permethrin-impregnated battle dress uniforms (BDU). Int J Hyg Environ Health.

[33] World Health Organization. Guidelines for laboratory and field testing of long-lasting insecticidal mosquito nets efficacy testing of mosquito repellents. 2005. https://apps.who.int/iris/bitstream/handle/10665/69007/WHO_CDS_WHOPES_GCDPP_2005.11.pdf;jsessionid=4C831F479B46E5F460E62EC389C865D6?sequence=1. Accessed 3 March 2021.

[34] Kawada H, Ohashi K, Dida GO, Sonye G, Njenga SM, Mwandawiro C, Minakawa N (2014). Insecticidal and repellent activities of pyrethroids to the three major pyrethroid-resistant malaria vectors in western Kenya. Parasites Vectors.

[35] World Health Organization (2004). WHO specifications and evaluations for public health pesticides—Icaridin.

[36] Licciardi S, Herve JP, Darriet F, Hougard J-M, Corbel V (2006). Lethal and behavioural effects of three synthetic repellents (DEET, IR3535 and KBR 3023) on *Aedes aegypti* mosquitoes in laboratory assays. Med Vet Entomol.

[37] Goodyer L, Schofield S (2018). Mosquito repellents for the traveler: does picaridin provide longer protection than DEET?. J Travel Med.

[38] Brown M, Hebert AA (1997). Insect repellents: an overview. J Am Acad Dermatol.

[39] Chareonviriyaphap T, Prabaripai A, Sungvornyothrin S (2002). An improved excito-repellency test chamber for mosquito behavioral tests. J Vect Ecol.

[40] Rutledge LC, Mehr ZA, Debboun M, By Debboun M, Frances SP, Strickman D (2015). Testing methods for insect repellents. Insect repellents handbook.

