# Supplementary figures and images for: New mosquito repellency bioassay for evaluation of repellents and pyrethroids using an attractive blood-feeding device
Source: Parasit Vectors. 2021 Mar 10;14:151. doi: 10.1186/s13071-021-04656-y (PMC7945690; doi:10.1186/s13071-021-04656-y)

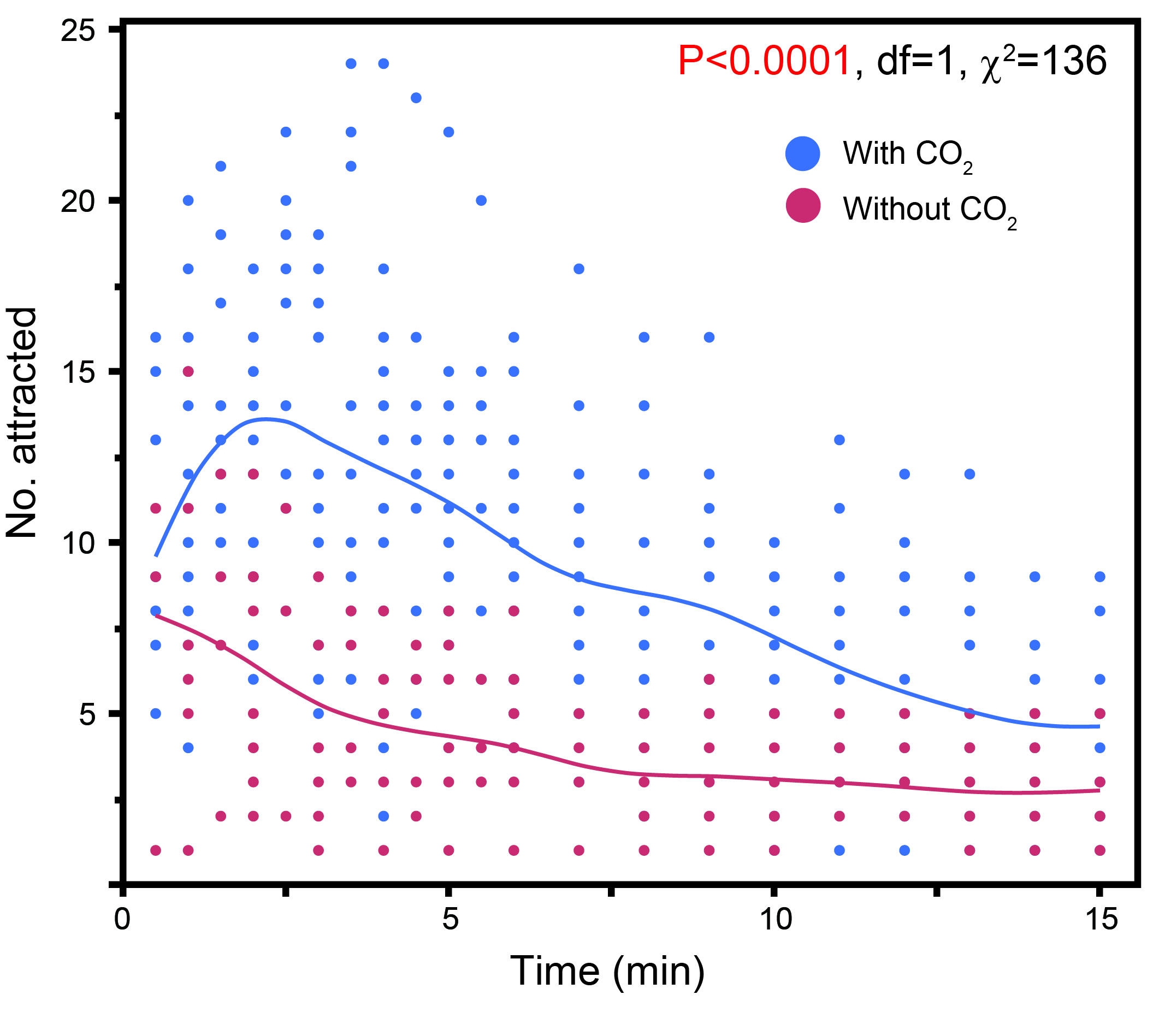

Supplement: Supplementary file 1 — Additional file 1: Figure S1. Effect of intermittently discharged carbon dioxide (10 s ON/50 s OFF, at the rate of 1.0 l/min) on the attraction of unfed Ae. albopictus females [file 13071_2021_4656_MOESM1_ESM.tif]

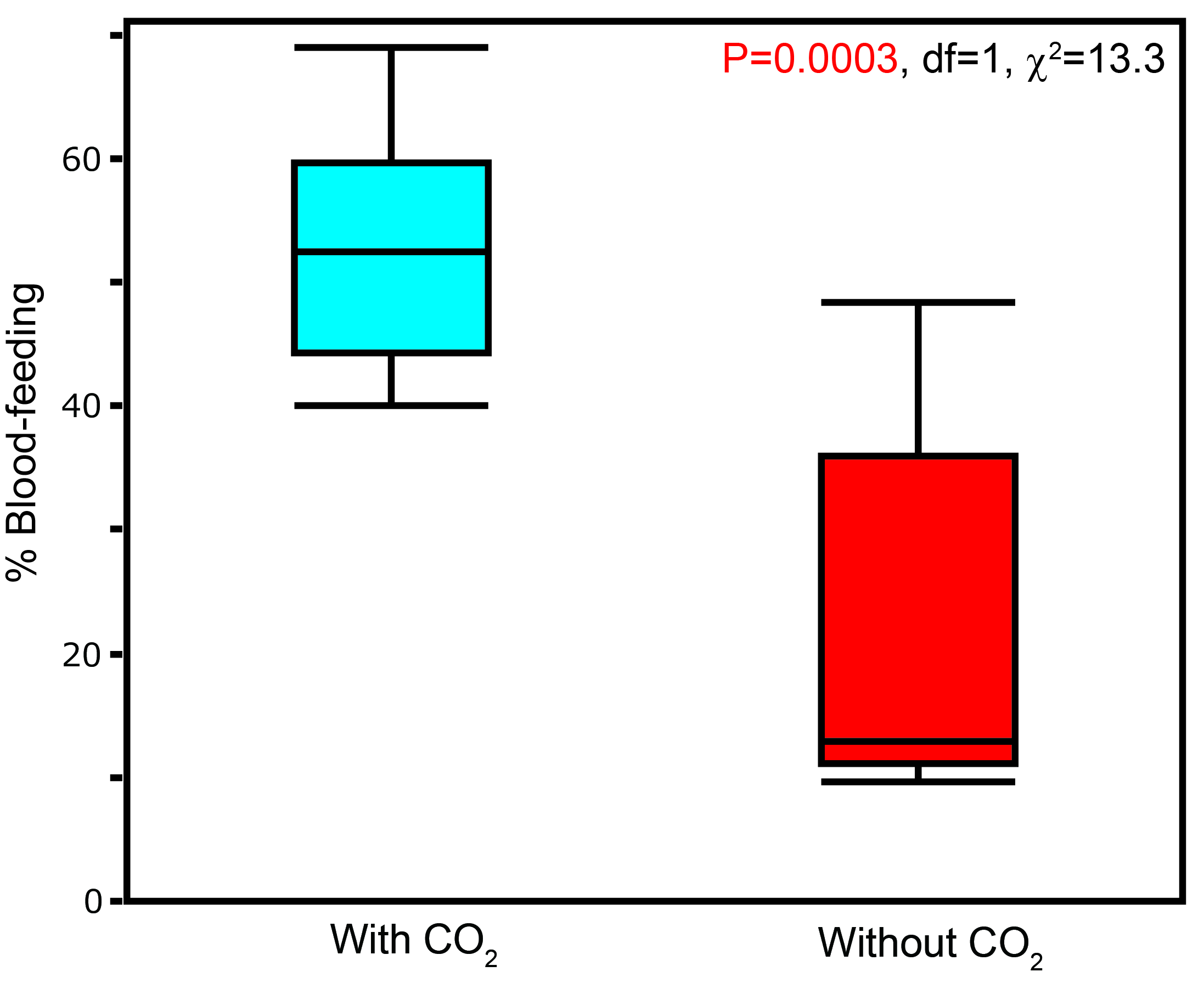

Supplement: Supplementary file 2 — Additional file 2: Figure S2. Effect of intermittently discharged carbon dioxide on the blood-feeding rate of unfed Ae. albopictus females [file 13071_2021_4656_MOESM2_ESM.tif]

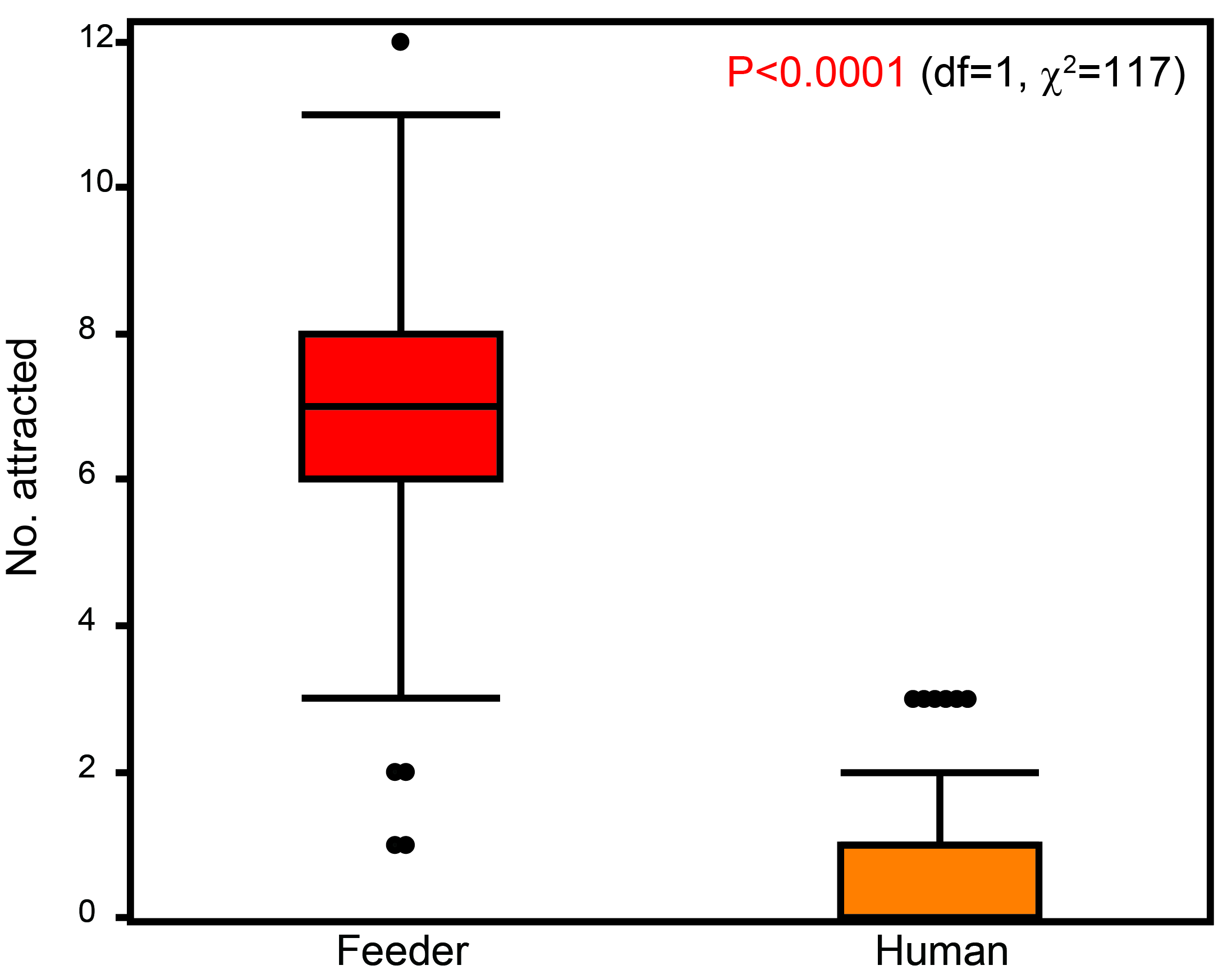

Supplement: Supplementary file 3 — Additional file 3: Figure S3. Difference in the attractiveness of the feeding units and human arm to unfed Ae. albopictus females under the same conditions using the test cage of ABFD [file 13071_2021_4656_MOESM3_ESM.tif]

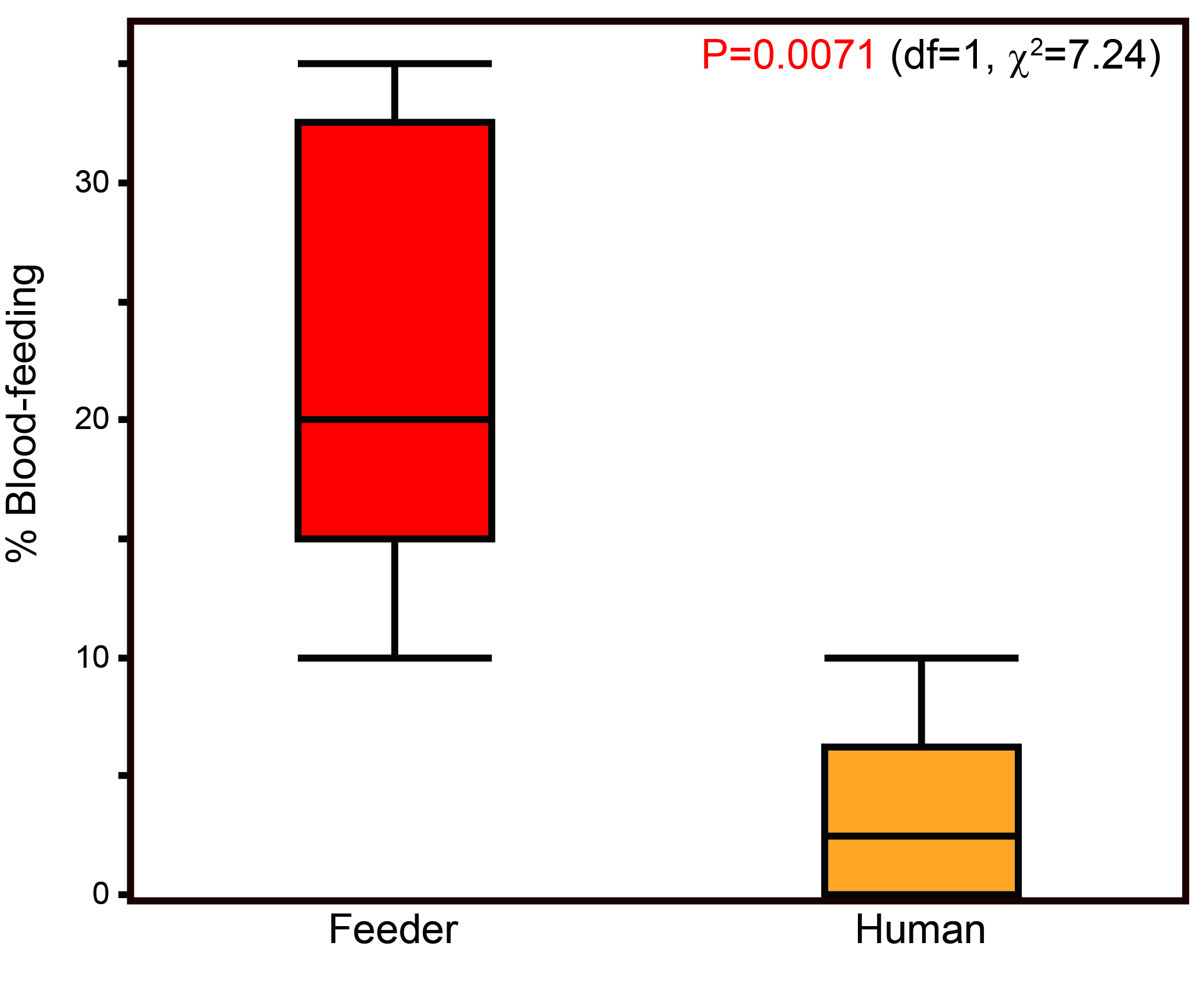

Supplement: Supplementary file 4 — Additional file 4: Figure S4. Difference in the blood-feeding rates of the feeding units and the human skin to unfed Ae. albopictus females under the same conditions using the test cage of ABFD [file 13071_2021_4656_MOESM4_ESM.tif]

## Slide 1
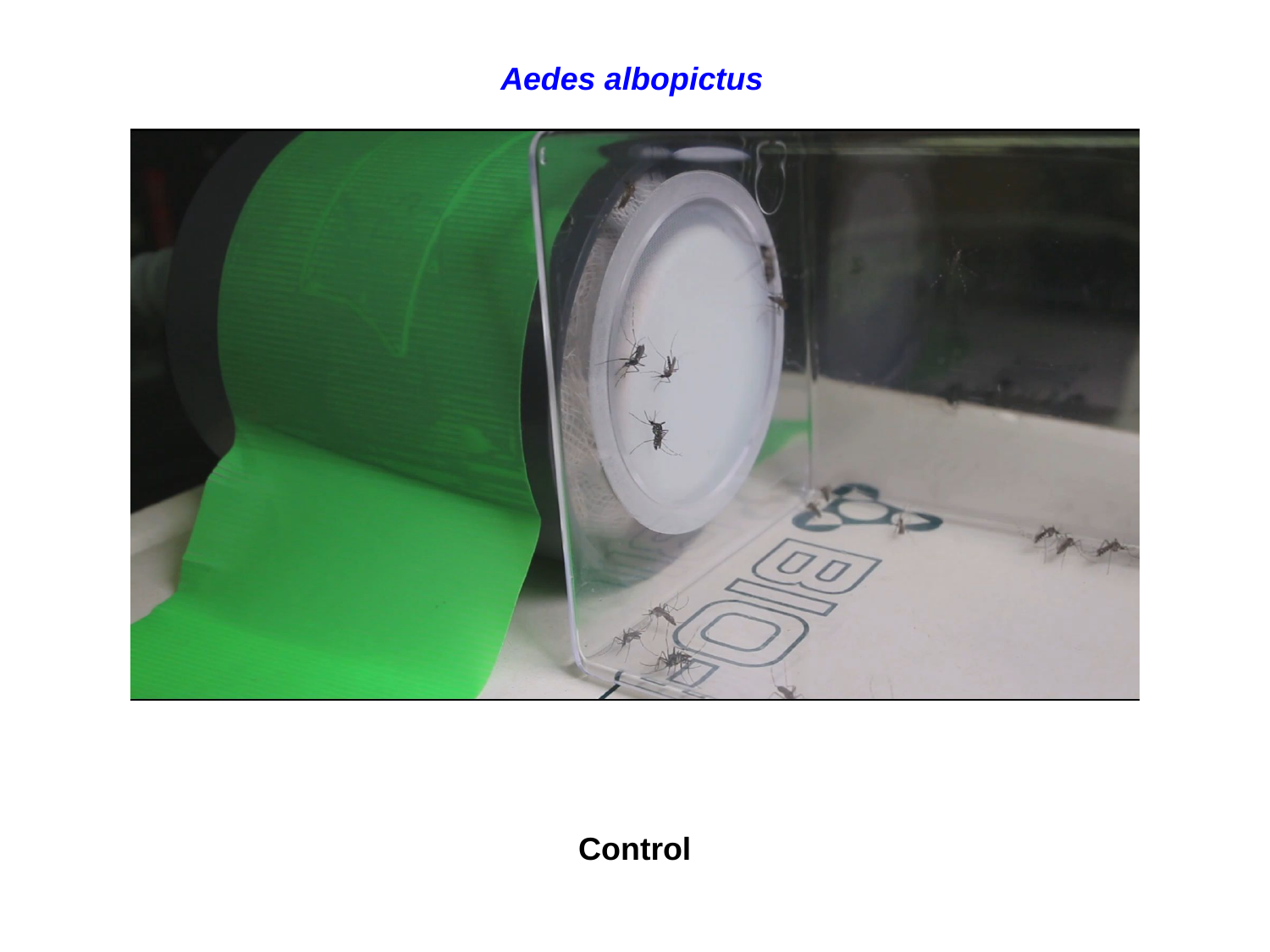

Aedes albopictus
Control

Supplement: Supplementary file 5 — Additional file 5: Video S1. Mosquito behavior on an untreated surface (PPTX 17854 KB) [file 13071_2021_4656_MOESM5_ESM.pptx]

## Slide 1
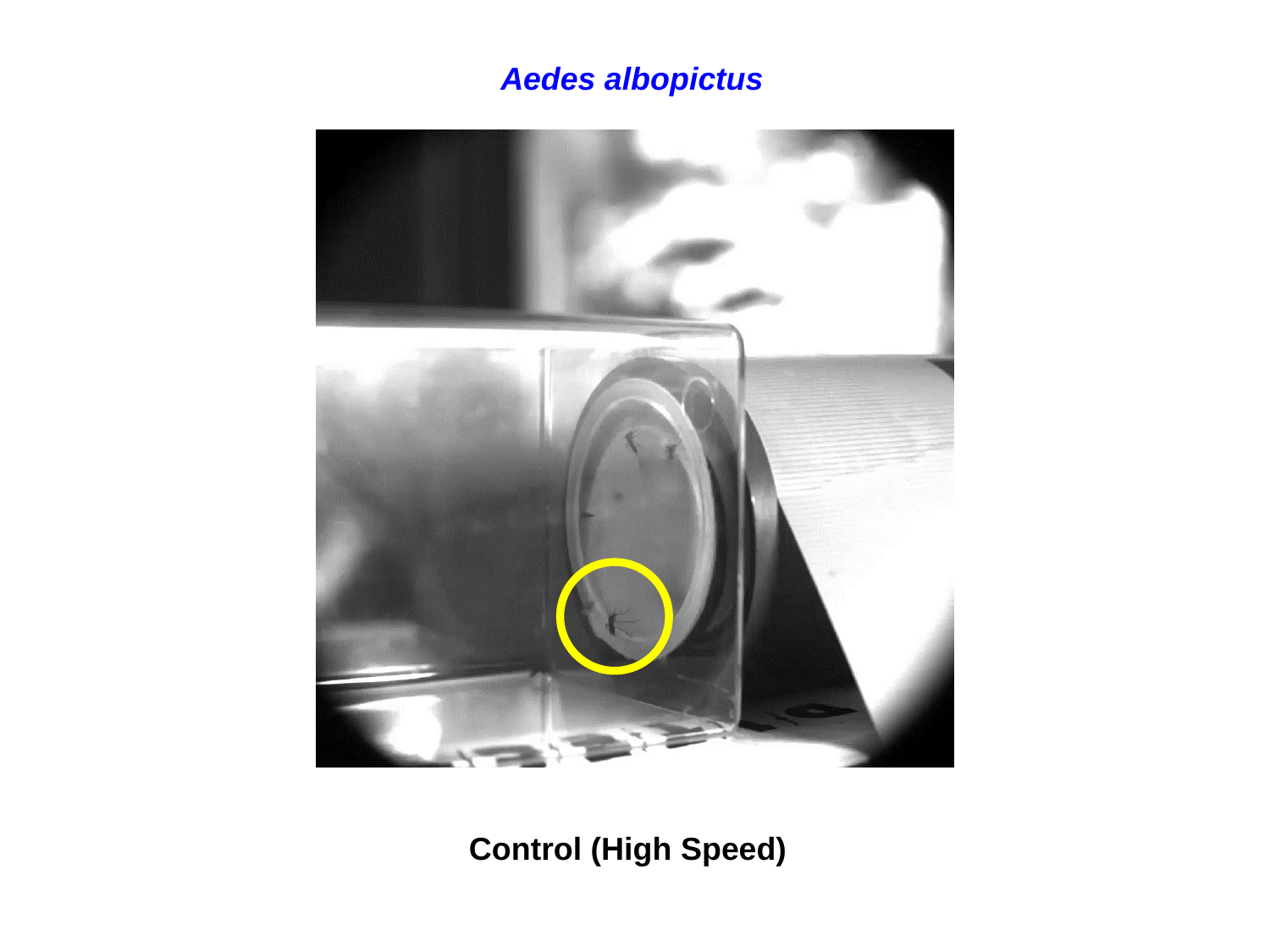

Aedes albopictus
Control (High Speed)

Supplement: Supplementary file 6 — Additional file 6: Video S2. Mosquito behavior on an untreated surface, registered by a high-speed camera [file 13071_2021_4656_MOESM6_ESM.pptx]

## Slide 1
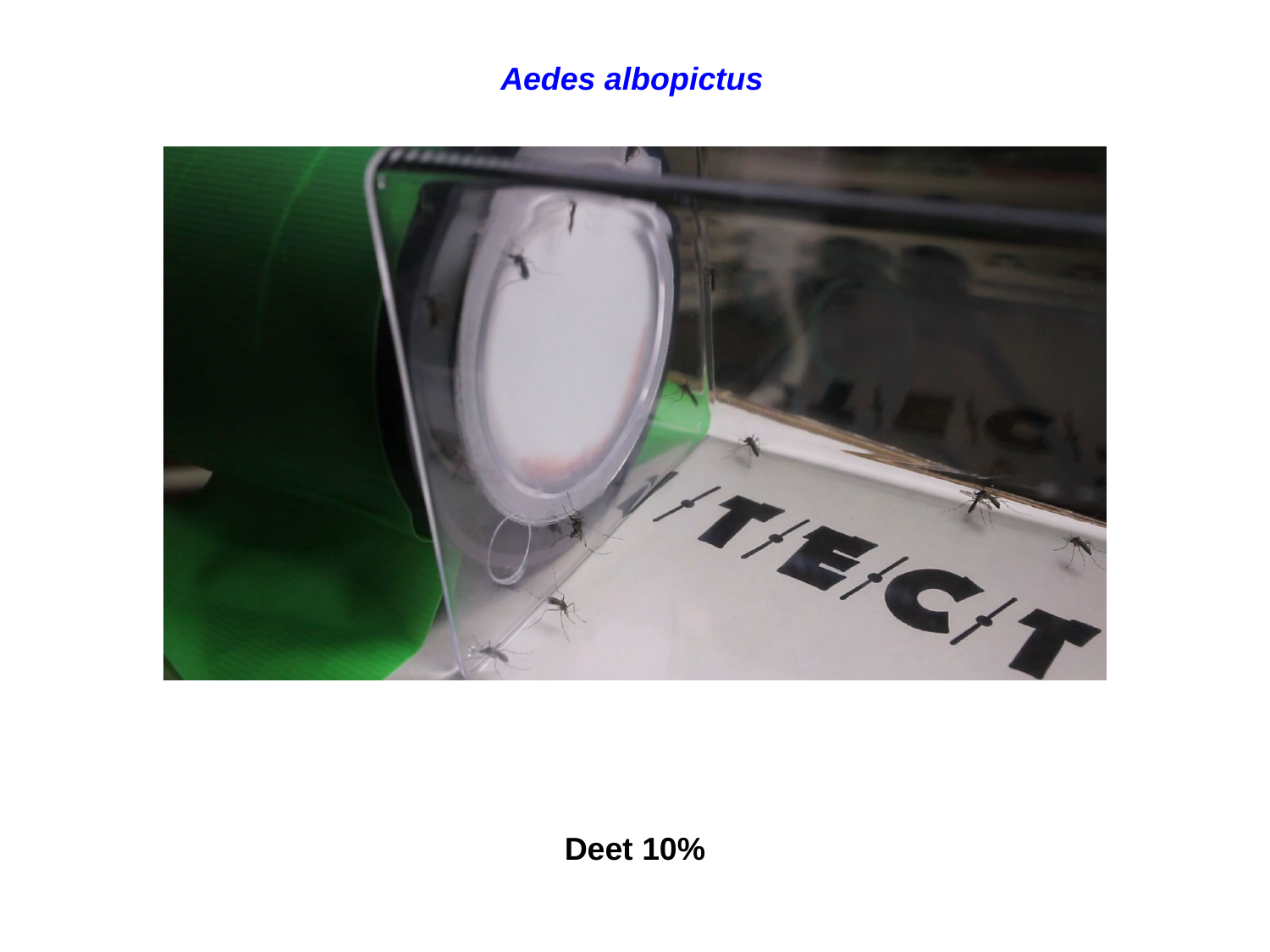

Aedes albopictus
Deet 10%

Supplement: Supplementary file 7 — Additional file 7: Video S3. Mosquito behavior on a surface treated with 10% DEET [file 13071_2021_4656_MOESM7_ESM.pptx]

## Slide 1
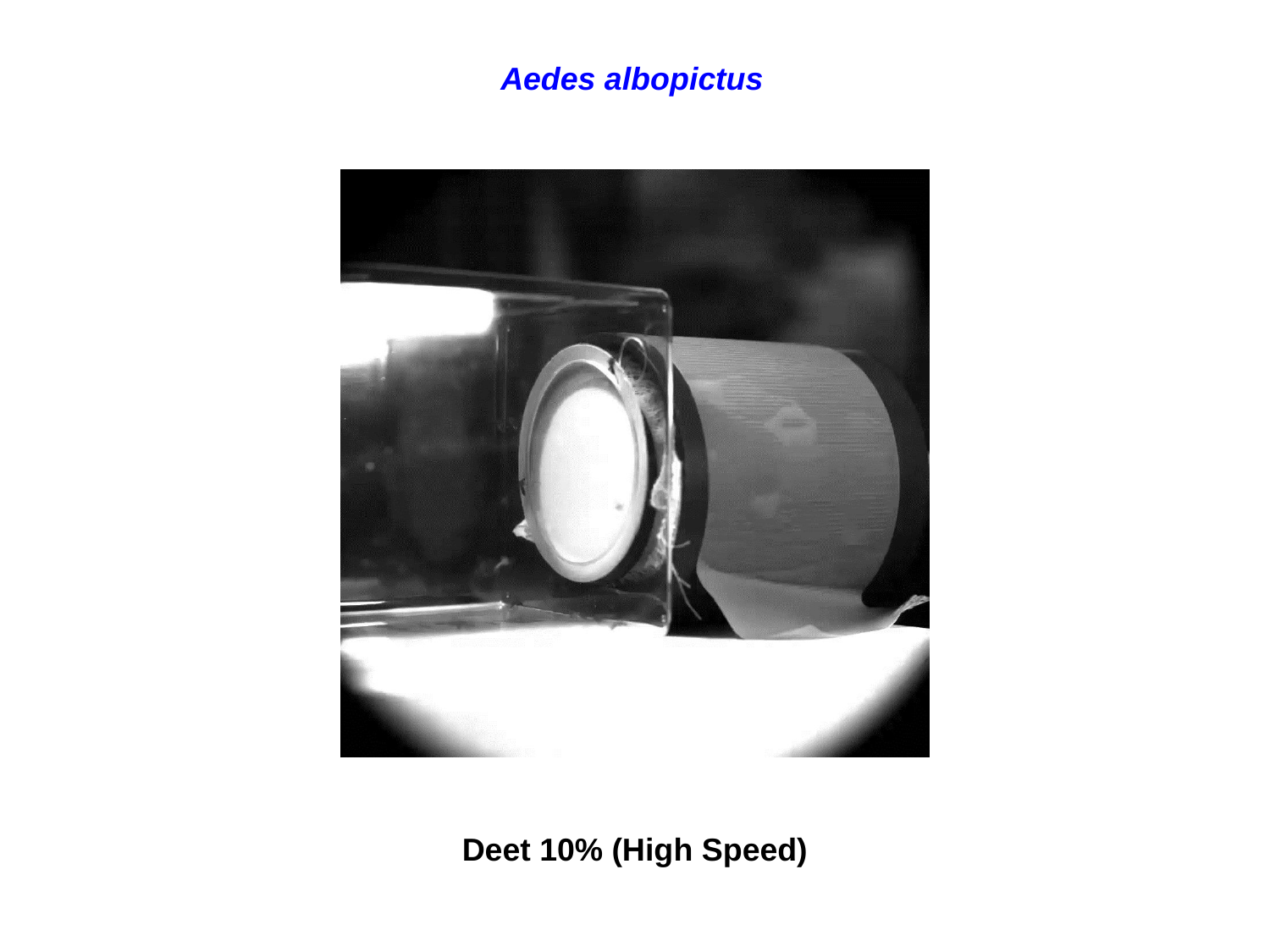

Aedes albopictus
Deet 10% (High Speed)

Supplement: Supplementary file 8 — Additional file 8: Video S4. Mosquito behavior on a surface treated with 10% DEET, registered by a high-speed camera [file 13071_2021_4656_MOESM8_ESM.pptx]

## Slide 1
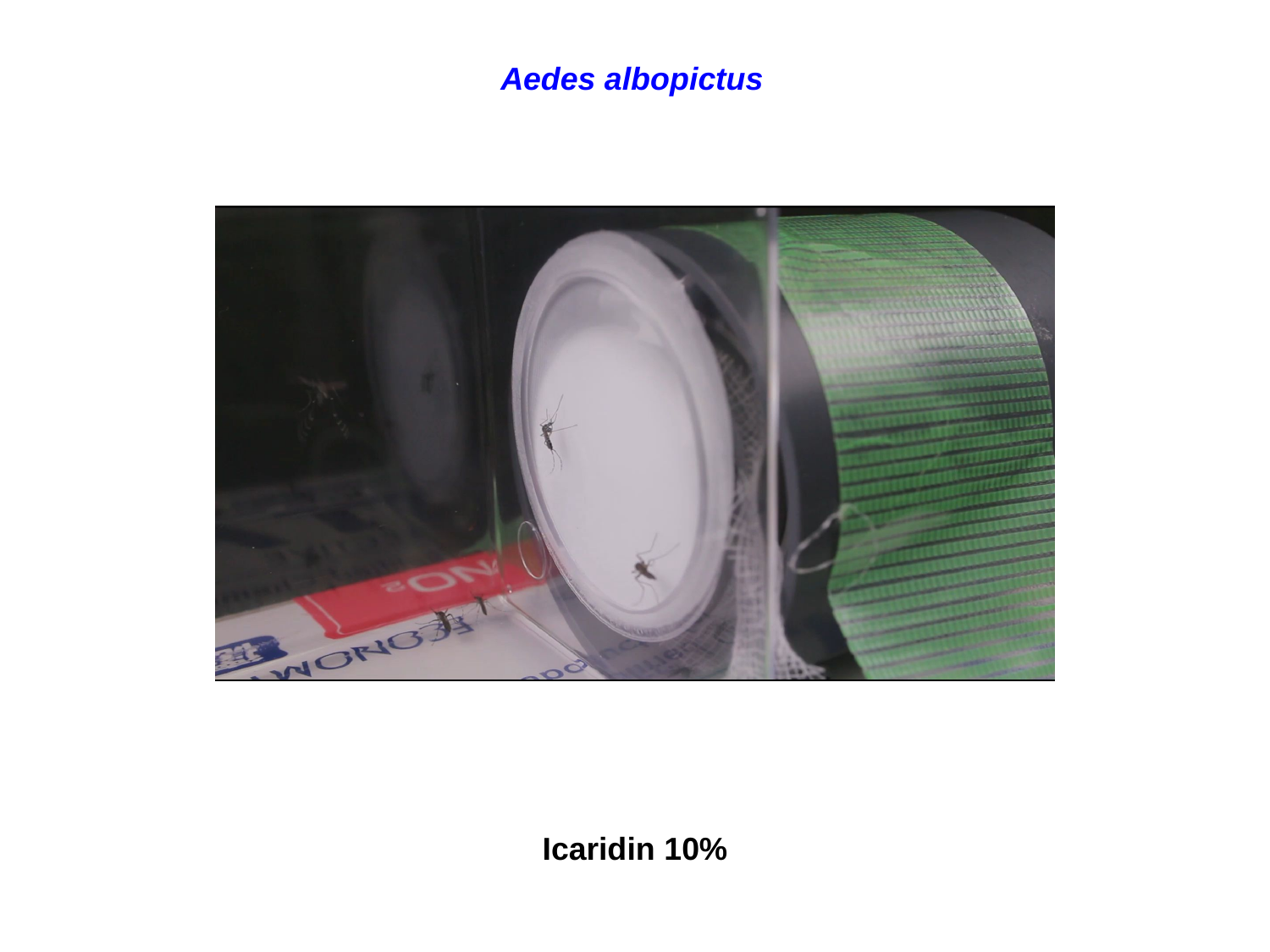

Aedes albopictus
Icaridin 10%

Supplement: Supplementary file 9 — Additional file 9: Video S5. Mosquito behavior on a surface treated with 10% icaridin [file 13071_2021_4656_MOESM9_ESM.pptx]

## Slide 1
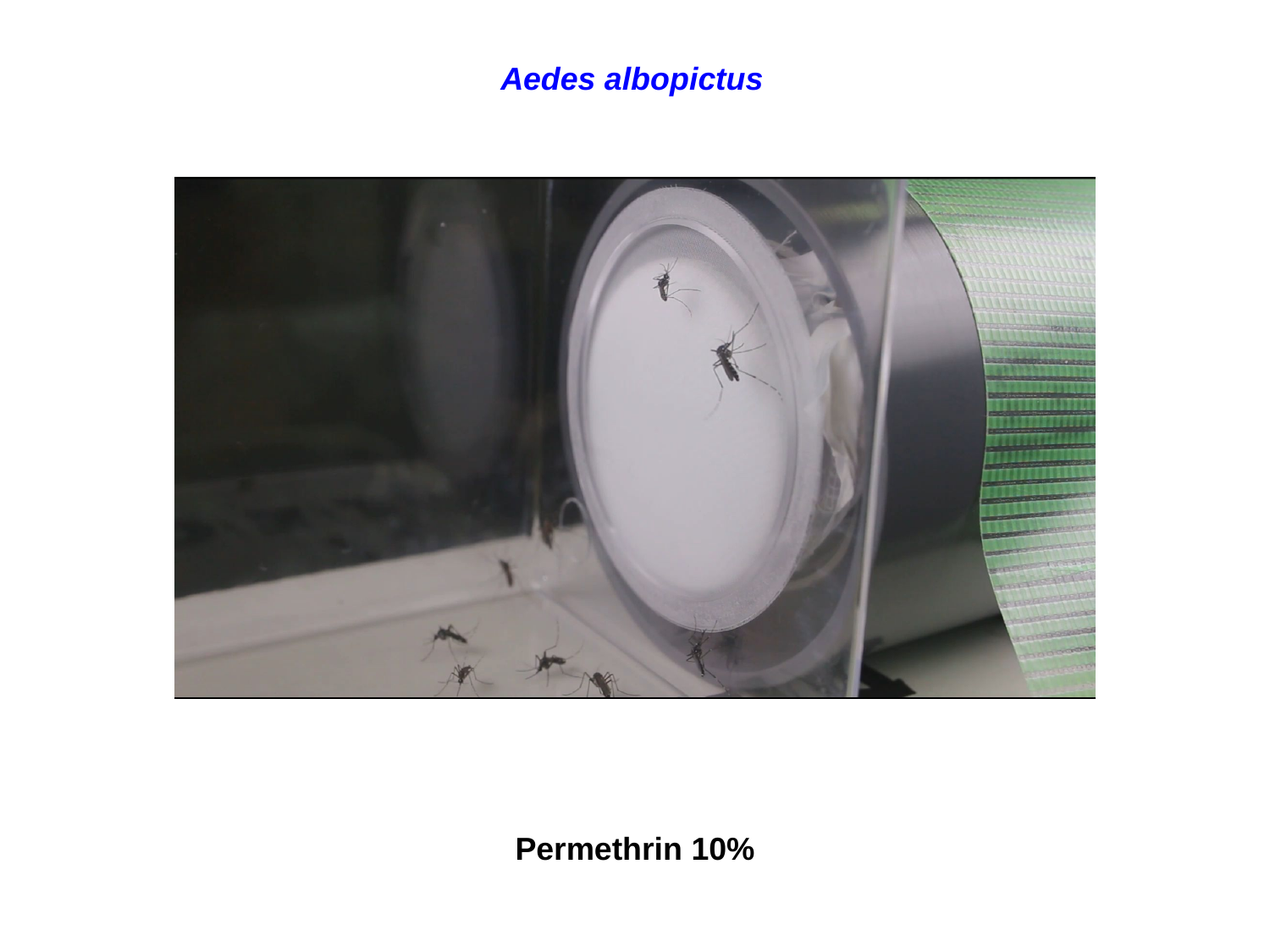

Aedes albopictus
Permethrin 10%

Supplement: Supplementary file 10 — Additional file 10: Video S6. Mosquito behavior on a surface treated with 10% permethrin [file 13071_2021_4656_MOESM10_ESM.pptx]
